# Preparation of Aluminosilicate Ferrierite Zeolite Nanosheets with Controllable Thickness in the Presence of a Sole Organic Structure Directing Agent

**DOI:** 10.3390/molecules25040771

**Published:** 2020-02-11

**Authors:** Hao Xu, YuXia Yu, LongFeng Zhu, ChaoQun Bian, HangLing Zhai, JianYing Tong, HuiZhen Wu, Chao Shen

**Affiliations:** 1 College of Biology and Environmental Engineering, Zhejiang Shuren University, Hangzhou 310015, China; xuhao@zju.edu.cn (H.X.); tjy132@msn.com (J.T.); huizhen1206@126.com (H.W.); 2 College of Biological, Chemical Sciences and Engineering, Jiaxing University, Jiaxing 314001, China; yyxx1012802885@163.com (Y.Y.); hangling1008@163.com (H.Z.); 3 Pharmaceutical and Material Engineering School, Jinhua Polytechnic, Jinhua 321000, China; bian00@zju.edu.cn

**Keywords:** aluminosilicate FER zeolite, morphology control, nanosheet, organic ammonium

## Abstract

Preparation of aluminosilicate ferrierite (FER) zeolite nanosheets with controllable thickness in the presence of a sole organic ammonium is attractive, but still challenging. In this report, with the employment of *N*,*N*-diethyl-*cis*-2,6-dimethylpiperidinium (DMP) as both a structure directing agent and crystal growth inhibitor, aluminosilicate FER zeolite nanosheets, with a variety of crystal thicknesses, ranging from 6 to 200 nm, are successfully synthesized under hydrothermal conditions. Very interestingly, the amount of DMP in the starting gel is the key factor for crystal thickness control of aluminosilicate FER zeolite nanosheets. The obtained FER products, with different thicknesses, are well characterized by X-ray powder diffraction (XRD), scanning electron microscopy (SEM), N_2_ sorption, thermogravimetric analysis (TG), inductively coupled plasma (ICP), and magic angle spinning nuclear magnetic resonance (MAS NMR) techniques. This simple strategy might provide a novel avenue for the synthesis of other zeolite nanosheets with controllable thickness.

## 1. Introduction

Zeolites, especially aluminosilicate zeolites, have been widely applied in the fields of adsorption, separation, ion exchange, and catalysis due to their uniform micropore distribution, large surface areas, and highly thermal and hydrothermal stabilities [1,2,3,4,5,6,7,8]. Currently, from the viewpoint of the chemistry of zeolites synthesis, synthesizing the novel structures, developing the novel synthesis methodology, and controlling the morphology of zeolites are the main directions [9,10,11,12,13,14]. Among them, controlling the morphology of zeolites is a hot topic because of their improved properties in the process of application [15,16,17,18,19,20,21]. For example, Ryoo et al. synthesized single-unit-cell nanosheets of Zeolite Socony Mobil-5 (ZSM-5) zeolite, which would facilitate diffusion, and thus avoid coke deposition significantly during methanol-to-gasoline conversion [22]. Xiao et al. reported that SAPO-11 nanosheets, with a thickness of 10–20 nm, exhibited higher selectivity for the isomers in the hydroisomerization of n-dodecane [23]. Li et al. prepared mordenite (MOR) zeolite nanosheets with good catalytic performance in the dimethyl ether (DME) carbonylation due to the large amount of Brønsted acidic sites and fast mass transfer [24].

FER zeolite is a medium-pore zeolite containing 10-Membered Ring (10-MR) channels (0.42 × 0.54 nm) in the [001] direction and 8-MR channels (0.35 × 0.48 nm) in the [010] direction [25,26,27,28,29,30,31]. Aluminosilicate FER zeolite, which is represented by ZSM-35, shows distinguished catalytic performances in many reactions, such as skeletal isomerization, high-olefin catalytic cracking, carbonylation of dimethyl ether, methanol to olefins, and NO_x_ reduction [25,26,32,33,34,35,36,37,38,39,40] To further enhance the catalytic performance, the preparation methods for aluminosilicate FER zeolites with controllable morphology and crystal sizes are particularly attractive. Corma et al. prepared FER nanocrystals with sizes of 10–20 nm using piperidine and surfactant as dual templates [25]. Meanwhile, FER nanoneedles with a diameter of 10 nm were developed by adding choline as an organic template [41]. Despite the fact that the diameter of the aluminosilicate FER zeolite crystals is very small in the above works, the crystal size could not be adjusted. Recently, Xu et al. reported on the preparation of sheet-like FER zeolite, with controllable thickness from 100 nm to 2 μm, using piperidine as a structure directing agent and cetyltrimethyl ammonium bromide (CTAB) as crystal growth inhibitors in one synthesis system [42]. However, the thinnest FER zeolite crystal in this work is still too thick and the dual template method is complicated. At present, it is still a great challenge to control the different nanosheet thicknesses of aluminosilicate FER zeolite within the thickness of 100 nm using the facile synthesis method.

More recently, we reported a simple method for synthesizing ultrathin nanosheets of aluminosilicate FER zeolite with the thickness of 6–8 nm by using a sole small organic ammonium (*N*,*N*-diethyl-*cis*-2,6-dimethylpiperidinium, DMP). Very interestingly, the DMP molecules not only direct the FER zeolite structure, but also inhibit the growth of FER zeolite on [100] direction, according to the theoretical calculation [43]. Therefore, it might give an opportunity for controlling the nanosheet thickness of aluminosilicate FER zeolite just by changing the amount of DMP molecules in the same synthesis system.

In this work, we report the synthesis of FER zeolite nanosheets with controllable thickness using a sole organic ammonium as both a structure directing agent and crystal growth inhibitor. Very interestingly, by adjusting the amount of DMP in the starting gel, the thickness of FER nanosheets can be adjusted, ranging from 6 to 200 nm.

## 2. Results and Discussion

**Figure 1:**
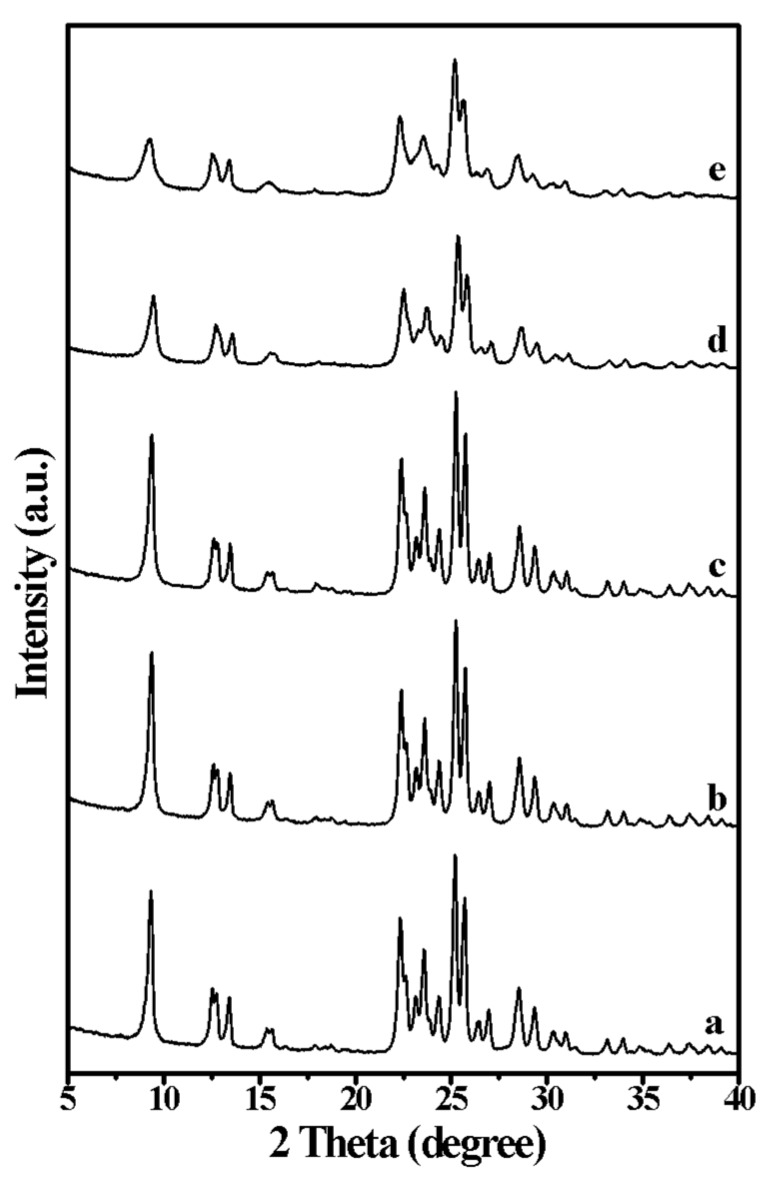
XRD patterns of the (a) FER-0, (b) FER-0.015, (c) FER-0.03, (d) FER-0.06, and (e) FER-0.12 samples, respectively.

**Figure 2:**
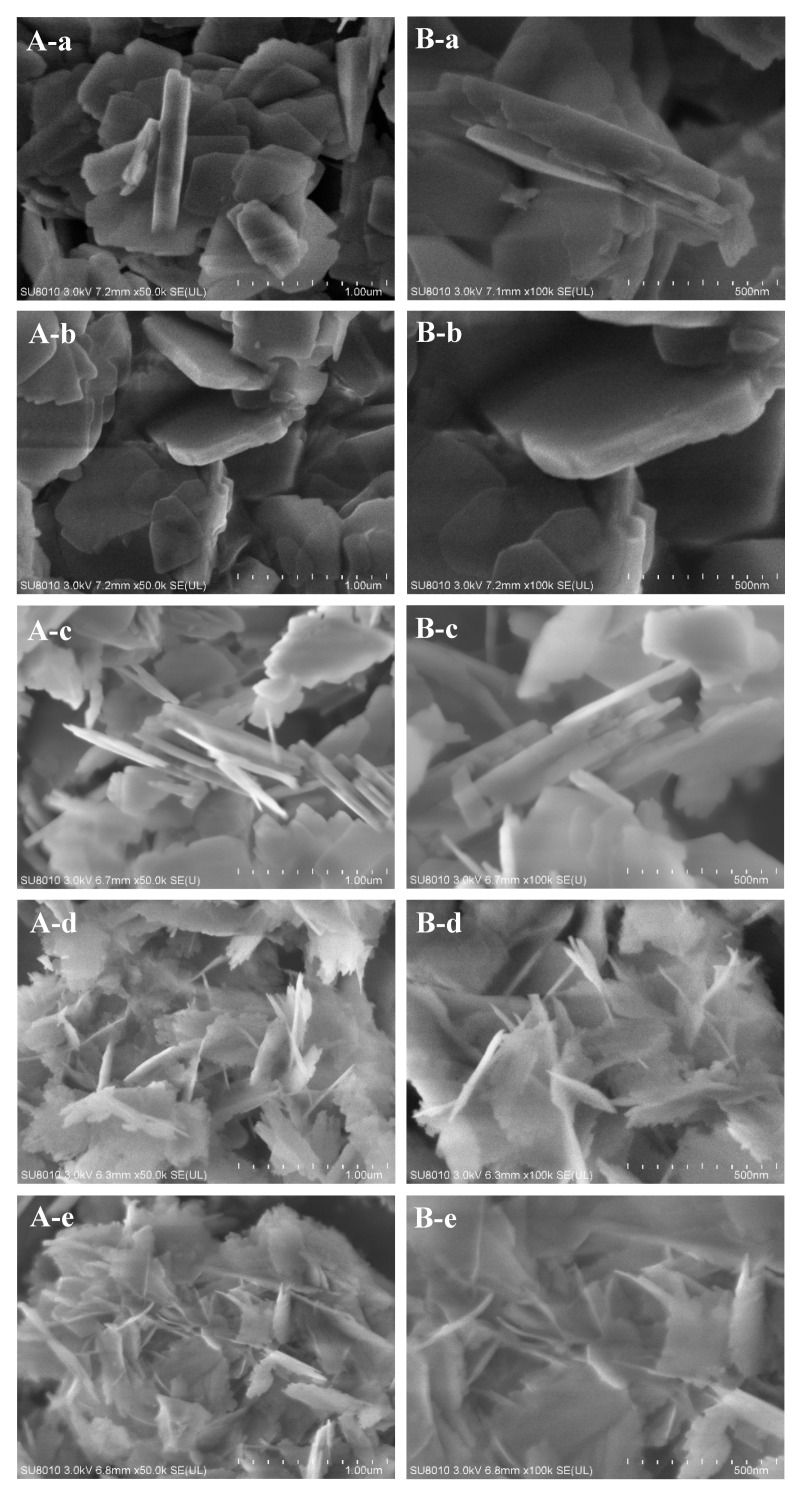
(**A**) Low-magnification and (**B**) high-magnification SEM images of the (**a**) FER-0, (**b**) FER-0.015, (**c**) FER-0.03, (**d**) FER-0.06 and (**e**) FER-0.12 samples, respectively.

Figure 1 shows the XRD patterns of aluminosilicate FER zeolite nanosheets with the different amount of DMP in the starting gel. Each XRD pattern of FER sample shows typical peaks of FER zeolite structure. The peaks at 9.3° associated with the [200] reflection widen with the increasing amount of DMP in the starting gel (Table 1), suggesting thinner thickness of FER zeolite along the [100] direction, which has been further confirmed by the results of the SEM images (Figure 2). According to the results of the SEM images in Figure 2 and sample synthesis conditions in Table 1, the thickness of FER-0 nanosheets is approximately 100–200 nm, without the addition of DMP in the starting gel (Figure 2a). When a small amount of DMP was added (DMP/SiO_2_ = 0.015) into the starting gel, the thickness of FER-0.015 nanosheets turned to around 50–100 nm (Figure 2b). Increasing the DMP/SiO_2_ ratio to 0.030, the nanosheets of FER-0.030 become thinner (30–60 nm, Figure 2c). Further increasing the DMP/SiO_2_ ratio to 0.060, the thickness of FER-0.06 nanosheets is around 10–20 nm (Figure 2d). Finally, when the DMP/SiO_2_ ratio reaches 0.12, the thinnest aluminosilicate FER zeolite nanosheets (FER-0.12) with a thickness of 6–8 nm would be obtained (Figure 2e) [43]. In addition, the SiO_2_/Al_2_O_3_ ratios of the FER zeolite samples with different thicknesses are all around 16.0–17.0 (Table 1), even if the amount of DMP molecules added in the synthesis gel is different. The above results show that the DMP molecules would inhibit the crystal growth of the aluminosilicate FER nanosheets, and thus obtain the different thickness of FER nanosheets from 6 to 200 nm.

**Figure 3:**
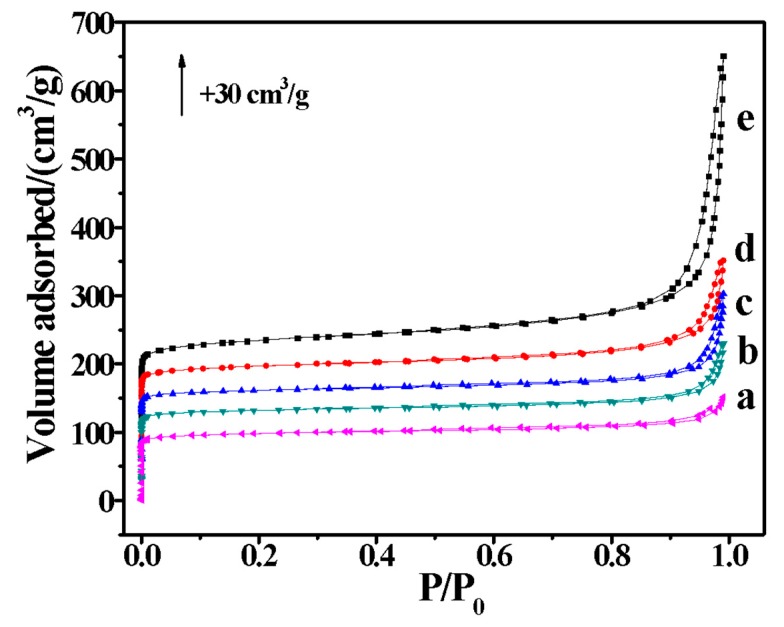
N_2_ sorption isotherms of the (a) H-FER-0, (b) H-FER-0.015, (c) H-FER-0.03, (d) H-FER-0.06, and (e) H-FER-0.12 samples, respectively.

Figure 3 shows nitrogen sorption isotherms of the aluminosilicate H-FER zeolite nanosheet samples. The micropore volumes of these samples are the same (0.14 cm^3^/g), while the mesopore volumes of these samples are increased with the decreasing of the crystal thickness, as shown in Table 2. Moreover, the external surface area and the Brunauer-Emmett-Teller (BET) urface area are higher when the crystal thickness of the aluminosilicate FER zeolite nanosheet samples decrease. The higher external surface area and BET surface area means a higher exposure degree, which is in good agreement with that of the zeolite nanosheets.

Figure S1 shows the TGA curves of the as-synthesized aluminosilicate FER zeolite samples, exhibiting different weight loss associated with the decomposition of organic structure directing agent (OSDA) in the micropores of the FER zeolite samples. This result shows that the DMP molecules are the structure directing agent for directing the generation of aluminosilicate FER zeolite. Very interestingly, the weight loss of the samples is very consistent with the DMP amount in the synthesis process.

**Figure 4:**
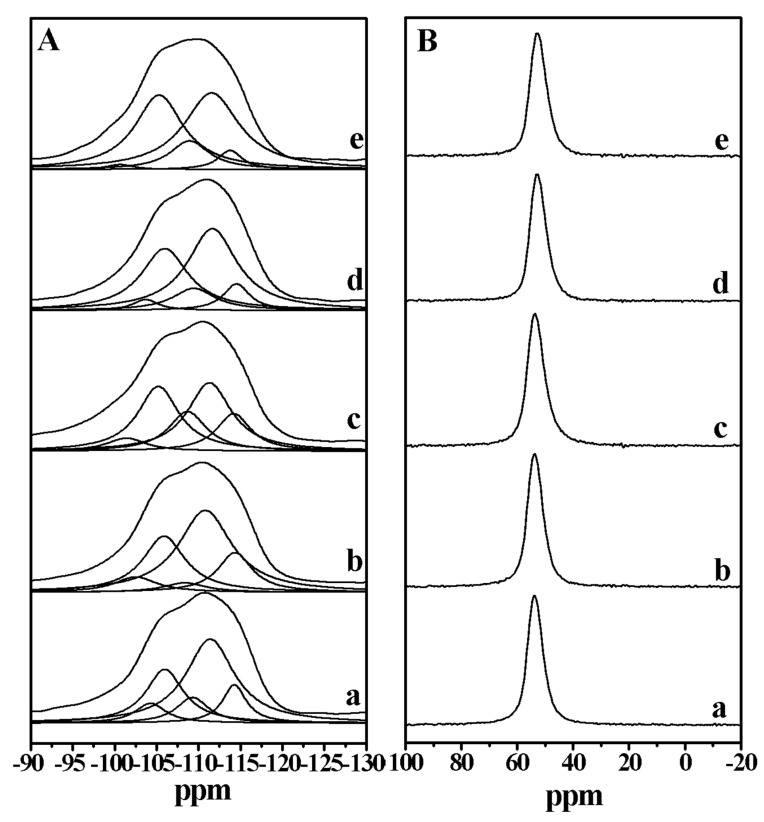
(**A**) ^29^Si magic angle spinning nuclear magnetic resonance (MAS NMR) and (**B**) ^27^Al MAS NMR spectra of (a) FER-0, (b) FER-0.015, (c) FER-0.03, (d) FER-0.06, and (e) FER-0.12 samples, respectively.

Figure 4A shows the ^29^Si magic angle spinning nuclear magnetic resonance (MAS NMR) spectra of the aluminosilicate FER zeolite samples. These samples exhibit peaks at around −114, −111, −109, −106, and −103 ppm, which can be reasonably assigned to Si(4Si) (−114, −111, and −109 ppm), Si(3Si) (−106 ppm), and Si(2Si) (−103 ppm) species, respectively. According to the spectra and the structural information in Table S1, the Si(4Si) and Si(2Si) species of the samples decrease along with the increasing amount of DMP in the process of synthesis, while the Si(3Si) species of the samples increases. This phenomenon might be the result from both the structure directing and growth inhibition effect of the DMP molecules. Figure 4B shows the ^27^Al MAS NMR spectra of the aluminosilicate FER zeolite samples. All of the samples give the one peak with the chemical shift at about 54 ppm associated with the tetrahedrally coordinated aluminum species in the FER zeolite framework. At the same time, there is no signal with the chemical shift at about zero ppm, suggesting the absence of extra-framework aluminum species in these samples. This result shows that the addition of different amounts of DMP molecules in the synthesis has no effect on the aluminum coordination of the samples.

The acidity of the aluminosilicate H-FER zeolite nanosheet samples is investigated by the NH_3_-TPD technique. The NH_3_-TPD curves in Figure S2 show that the samples with thinner crystal thickness have fewer acidic sites. This might be caused by the growth inhibition effect of the DMP molecules, which is in good agreement with the literature [42].

## 3. Materials and Methods

### 3.1. Starting Materials

Sodium metaaluminate (NaAlO_2_, AR, 99%, Sinopharm Chemical Reagent Co., Ltd., Shanghai, China), sodium hydroxide (NaOH, AR, 96%, Sinopharm Chemical Reagent Co., Ltd., Shanghai, China), colloidal silica (40 wt% SiO_2_ in water, Sigma-Aldrich Reagent Co., Ltd., MO, USA), *cis*-2,6-dimethylpiperidine (Sigma-Aldrich Reagent Co., Ltd., MO, USA), potassium bicarbonate (KHCO_3_, AR, 99.5%, Sinopharm Chemical Reagent Co., Ltd., Shanghai, China), iodoethane (99%, Aladdin Chemical Co., Ltd., Shanghai, China), methanol (Sinopharm Chemical Reagent Co., Ltd., Shanghai, China), diethyl ether (AR, 99.5%, Sinopharm Chemical Reagent Co., Ltd., Shanghai, China), anion-exchange resin (Amberlite IRN-78, OH-form, Thermofisher Chemical Reagent Co., Ltd., CA, USA), and ammonium nitrate (NH_4_NO_3_, AR, 99%, Beijing Chemical Reagent Co., Ltd., Beijing, China) were used without further purification. The deionized water was homemade.

### 3.2. Synthesis of OSDA

The iodide form of the OSDA, *N*,*N*-diethyl-*cis*-2,6-dimethylpiperidine iodide was synthesized by mixing *cis*-2,6-dimethylpiperidine, iodoethane, and KHCO_3_ in the methanol, followed by refluxing for 4 days. The excess of KHCO_3_ was removed, and then the solvent and the excess iodoethane was evaporated. Next, the product was washed with diethyl ether and converted to the hydroxide form using an anion exchange resin.

### 3.3. Synthesis of Aluminosilicate FER Zeolite Nanosheets

In a typical run for synthesizing aluminosilicate FER zeolite nanosheets without the addition of DMP molecules, 0.165 g of NaAlO_2_ and 0.254 g of NaOH was dissolved into deionized water. Then 2.95 g of colloidal silica (40 wt% SiO_2_ in water) was added and the mixture was stirred for 2 h. Next, 0.024 g of FER zeolite seeds was added. Finally, the mixture was transferred into a Teflon-lined autoclave oven and crystallized at 140 °C for 48 h under rotation conditions (50 rpm). After filtering, washing, and drying, the product was gained.

In a typical run for synthesizing aluminosilicate FER zeolite nanosheets with the use of DMP, 0.165 g of NaAlO_2_ and 0.254 g of NaOH was dissolved into deionized water. Next, a certain amount of DMP solution and 2.95 g of colloidal silica (40 wt% SiO_2_ in water) were added. After stirring for 2 h, the mixture was transferred into a Teflon-lined autoclave oven and crystallized at 140 °C for 48 h under rotation conditions (50 rpm). After filtering, washing, and drying, the products were gained.

The as-made form of the products were denoted as FER-x (x represent the ratio of DMP/SiO_2_ in the starting gel). The H-form of the samples (H-FER-x) were obtained by calcining and ion-exchanging the FER-x samples with the NH_4_NO_3_ solution.

### 3.4. Methods

X-ray powder diffraction (XRD) patterns were measured with a Rigaku Ultimate VI X-ray diffractometer (40 kV, 40 mA) using Cu_Kα_ (λ = 1.5406 Å) radiation. Scanning electron microscopy (SEM) experiments were performed on Hitachi SU-8010 electron microscopes. The N_2_ sorption isotherms at the temperature of liquid nitrogen were measured using Micromeritics ASAP 2020M and Tristar system. The thermogravimetric analysis (TGA) experiments were carried out on a Perkin-Elmer TGA 7 unit in air, at a heating rate of 10 °C/min, in the temperature range from room temperature to 800 °C. ^29^Si and ^27^Al MAS NMR spectra were recorded on a Varian Infinity Plus 400 spectrometer. The sample composition was determined by inductively coupled plasma (ICP) with a Perkin-Elmer 3300DV emission spectrometer. The acidity of the samples was measured by the temperature-programmed-desorption of ammonia (NH_3_-TPD). The 50 mg of samples were placed in a quartz tube and pretreated in He flow at 600 °C for 30 min. Then the temperature was reduced to 150 °C. NH_3_ passed through the samples until it reached equilibrium for 30 min. When the baseline was stable, the signal of NH_3_ desorption was monitored by the thermal conductivity detector (TCD) in He flow at a heating rate of 20 °C/min from 150 to 650 °C.

## 4. Conclusions

In summary, aluminosilicate FER zeolite nanosheets with controllable thickness are successfully prepared with the use of a sole small organic ammonium (DMP). The addition of different amounts of DMP molecules in the starting gel is the key factor for successful control of the FER nanosheet crystal thickness. All of the nanosheets of the FER zeolite samples have good crystallinity, uniform morphology, large BET surface area, four-coordinated aluminum species, and abundant acidic sites. This simple synthesis strategy might be applied when it comes to preparing, and improving the performance, of other zeolite nanosheets with controllable thicknesses.

## Figures and Tables

**Table 1:** Synthesis of nanosheets of FER zeolite under various conditions.

Run	SiO_2_/Al_2_O_3_	Na_2_O/SiO_2_	DMP/SiO_2_	Seeds/SiO_2_	Products	Thickness/nm	SiO_2_/Al_2_O_3_ of the Product
1	28.0	0.20	0	0.02	FER-0	100–200	17.0
2	28.0	0.20	0.015	0	FER-0.015	50–100	16.6
3	28.0	0.20	0.030	0	FER-0.03	30–60	16.3
4	28.0	0.20	0.060	0	FER-0.06	10–20	16.5
5	28.0	0.20	0.12	0	FER-0.12	6–8	16.0

**Table 2:** Textural parameters of the aluminosilicate FER zeolite samples.

Sample	S_BET_ (m^2^/g)	S_micro_ (m^2^/g)	S_ext_ (m^2^/g)	V_tot_ (cm^3^/g)	V_micro_ (cm^3^/g)	V_meso_ (cm^3^/g)
H-FER-0	326	303	23	0.23	0.14	0.09
H-FER-0.125	355	319	36	0.29	0.14	0.15
H-FER-0.25	367	327	40	0.35	0.14	0.21
H-FER-0.5	377	318	59	0.39	0.14	0.25
H-FER-1.0	391	286	105	0.79	0.14	0.65

## Supplementary Materials

The following are available online, Figure S1: TGA curves of the (a) FER-0, (b) FER-0.015, (c) FER-0.03, (d) FER-0.06, and (e) FER-0.12 samples, respectively, Figure S2: NH_3_-TPD curves of the (a) H-FER-0, (b) H-FER-0.015, (c) H-FER-0.03, (d) H-FER-0.06, and (e) H-FER-0.12 samples, respectively, Table S1: Structural information on FER zeolite samples from ^29^Si NMR analysis.
