# Supplementary material for: Preparation of Aluminosilicate Ferrierite Zeolite Nanosheets with Controllable Thickness in the Presence of a Sole Organic Structure Directing Agent
Source: Molecules. 2020 Feb 11;25(4):771. doi: 10.3390/molecules25040771 (PMC7070331; doi:10.3390/molecules25040771)
Supplement: Supplementary file 1 [file molecules-25-00771-s001.pdf]

Supporting Information

Preparation of Aluminosilicate Ferrierite Zeolite Nanosheets with Controllable Thickness in the Presence of a Sole Organic Structure Directing Agent

Hao Xu [1], Yuxia Yu [2], Longfeng Zhu [2,*], Chaoqun Bian [3], Hangling Zhai [2], Jianying Tong [1], Huizhen Wu [1], and Chao Shen [1,*]

[1] College of Biology and Environmental Engineering, Zhejiang Shuren University, Hangzhou 310015, P. R. China;
[2] College of Biological, Chemical Sciences and Engineering, Jiaxing University, Jiaxing 314001, P. R. China;
[3] Pharmaceutical and Material Engineering School, Jinhua Polytechnic, Jinhua 321000, P. R. China.
* Correspondence: zhulf1988@mail.zjxu.edu.cn (L.F.Z); Tel.: +86-573-8364-0131; shenchaozju@zjsru.edu.cn (C. S.); Tel.: +86-571- 8829-7172

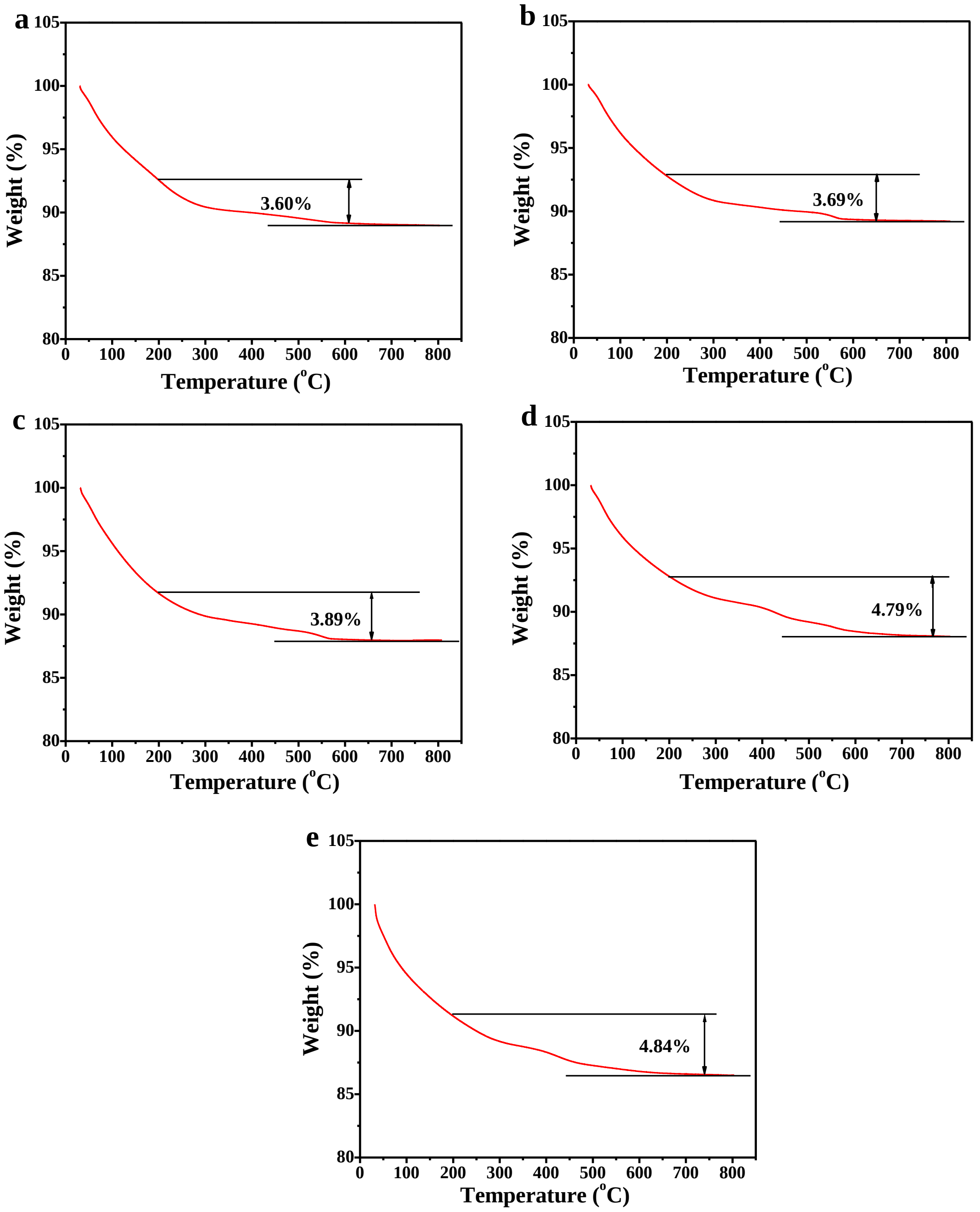

Figure S1. TG curves of the (a) FER-0, (b) FER-0.015, (c) FER-0.03, (d) FER-0.06 and (e) FER-0.12 samples, respectively.

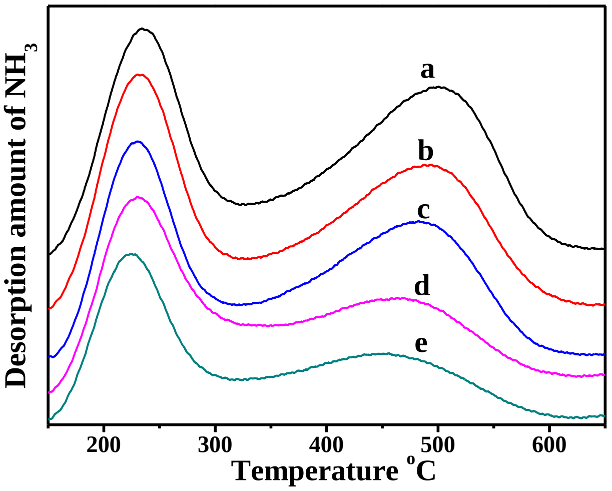

Figure S2. NH_3-TPD curves of the (a) H-FER-0, (b) H-FER-0.015, (c) H-FER-0.03, (d) H-FER-0.06 and (e) H-FER-0.12 samples, respectively.

Table S1. Structural information on FER zeolite samples from ^{29}Si NMR analysis.

Sample	Si(4Si)/%	Si(3Si)/%
FER-0	68.7	31.3
FER-0.125	66.9	33.1
FER-0.25	64.4	35.6
FER-0.5	64.1	35.9
FER-1.0	61.0	39.0
